# Defining within-host SARS-CoV-2 RNA viral load kinetics during acute COVID-19 infection within different respiratory compartments and their respective associations with host infectiousness: a protocol for a systematic review and meta-analysis

**DOI:** 10.1136/bmjopen-2024-085127

**Published:** 2024-12-02

**Authors:** Daniel Pan, Christopher A Martin, Joshua Nazareth, Shirley Sze, Amani Al-Oraibi, Mayuri Gogoi, Natalia Grolmusova, Pip Divall, Jonathan Decker, Eve Fletcher, Caroline Williams, James Hay, Rebecca F. Baggaley, Anne L Wyllie, Iain Stephenson, Erol Gaillard, Laura B Nellums, Tristan William Clark, Jonathan Nguyen Van-Tam, Benjamin J Cowling, T. Déirdre Hollingsworth, Laura Gray, Michael Barer, Manish Pareek

**Affiliations:** 1 Development Centre for Population Health, University of Leicester, Leicester, UK; 2 Department of Infectious Diseases and HIV Medicine, University Hospitals of Leicester NHS Trust, Leicester, UK; 3 Department of Respiratory Sciences, University of Leicester, Leicester, UK; 4 Leicester NIHR Biomedical Research Centre, Leicester, UK; 5 Oxford Big Data Institute, University of Oxford, Oxford, UK; 6 WHO Collaborating Centre for Infectious Disease Epidemiology and Control, Hong Kong School of Public Health, Hong Kong, Hong Kong; 7 Department of Cardiovascular Sciences, University of Leicester, Leicester, UK; 8 Lifespan and Population Health, University of Nottingham, Nottingham, UK; 9 Education Centre Library, University Hospitals of Leicester NHS Trust, Leicester, UK; 10 Department of Clinical Microbiology, University Hospitals of Leicester NHS Trust, Leicester, UK; 11 Department of Epidemiology of Microbial Diseases, Yale University, New Haven, Connecticut, USA; 12 Department of Paediatric Respiratory Medicine, University Hospitals of Leicester NHS Trust, Leicester, UK; 13 College of Population Health, University of New Mexico Health Sciences Center, Albuquerque, New Mexico, USA; 14 Clinical and Experimental Sciences, University of Southampton, Southampton, UK; 15 Department of Infection, University Hospital Southampton NHS Foundation Trust, Southampton, UK; 16 NIHR Southampton Biomedical Research Centre, Southampton, UK; 17 Laboratory of Data Discovery for Health Limited, Hong Kong Science and Technology Parks Corporation, Hong Kong, Hong Kong; 18 NDM Centre for Global Health Research, Nuffield Department of Medicine, University of Oxford, Oxford, UK; 19 Department of Population Health Sciences, University of Leicester, Leicester, UK; 20 NIHR Applied Research Collaborative East Midlands, Leicester, UK

**Keywords:** Infection control, INFECTIOUS DISEASES, Meta-Analysis, RESPIRATORY MEDICINE (see Thoracic Medicine), Review

## Abstract

**Abstract:**

**Introduction:**

Understanding how RNA viral load changes (viral load kinetics) during acute infection in SARS-CoV-2 can help to identify when and which patients are most infectious. We seek to summarise existing data on the longitudinal RNA viral load kinetics of SARS-CoV-2 sampled from different parts of the respiratory tract (nose, nasopharynx, oropharynx, saliva and exhaled breath) and how this may vary with age, sex, ethnicity, immune status, disease severity, vaccination, treatment and virus variant.

**Methods and analysis:**

We will conduct a systematic review and meta-analysis, using studies identified through MEDLINE and EMBASE (via Ovid). All research studies reporting primary data on longitudinal RNA viral load kinetics of infected patients with SARS-CoV-2 will be included. Methodological quality will be assessed using a validated checklist for longitudinal studies as well as predefined quality criteria for assessment of individual-level RNA viral load. Should the data allow, we will aim to perform individual patient-level meta-analysis. Our primary outcomes are duration to, and quantity of peak RNA viral load, and total duration of viral load shedding within different respiratory compartments. Secondary outcomes include duration of lateral flow antigen and virus culture positivity and variation of RNA viral load by age, immune status, disease severity, vaccination, treatment, lateral flow tests, viral culture positivity and SARS-CoV-2 variant. Study-level effects affecting observations, but not related to properties of the patient, such as the PCR platform and gene target will also be recorded. Random-effects models will estimate the population mean and individual-level variation in viral shedding conditional on the aforementioned variables. Finally, we will summarise the key mechanistic models used in the literature to reconstruct individual-level viral kinetics and estimate how different factors shape viral dynamics over time.

**Ethics and dissemination:**

Ethical approval is not needed as data will be obtained from published articles or studies with data that have already received and ethical review for analysis. Manuscript(s) will be prepared for publication.

**Systematic review protocol registration:**

PROSPERO ID: CRD42023385315

**STRENGTHS AND LIMITATIONS OF THIS STUDY**

- Our protocol is comprehensive in nature and addresses an important public health question relating to viral load kinetics from different parts of the respiratory tract.
- Limitations include a lack of granular covariate data in relation to individual RNA viral load kinetics presented in primary research may be a barrier to exploring how RNA viral load kinetics may vary with different subgroups (eg, vaccinated, non-vaccinated and different variants).
- Different studies may also use different triggering events to begin testing (exposure, prospective testing, symptom onset, first contact with medical care), which makes aligning the timing of the viral kinetics very difficult (but modelling approaches will be presented to deal with this).

## Introduction

 Community transmission of SARS-CoV-2 continues to occur in populations with high levels of immunity.^1^ Successive SARS-CoV-2 variants of concern (VOCs) have demonstrated an increase in epidemic growth rates.^2^,^4^ Specific real-time quantitative reverse transcription polymerase chain reaction (RT-qPCR) is used to SARS-CoV-2 infection COVID-19 and can be collected from a variety of different respiratory samples, most commonly the nasopharynx. While this is highly sensitive and specific, detection of virus RNA itself does not distinguish between replication-competent virus and residual RNA. Infectiousness currently is often established using one of four proxies: presence of virus RNA above a defined cycle threshold (Ct) value or genome copy number, positive rapid antigen test, isolation of replication-competent virus or proof of transmission to another person.

Over the course of infection, RNA viral load measured within different respiratory compartments, most commonly the nasopharynx, increases after infection, reaches a peak and then declines with subsequent virus clearance. How RNA viral load changes over time, during the course of their acute infection, is known as viral load kinetics. Identification of how long people infected with SARS-CoV-2 shed viable virus, as well as the time to and quantity of their peak viral load, is key to developing a better understanding of host infectiousness and, thus, the development of effective infection control policies. Viral load kinetics may also vary between individuals due to host factors such as age, sex, prior immunity or genetic changes to SARS-CoV-2 in relation to the emergence of new variants. Understanding how these factors influence viral load kinetics and in turn the association of viral load with infectiousness is crucial for developing up-to-date infection prevention control guidance.

Previous studies have investigated within-host viral load kinetics with disease severity, VOC and infectivity.^15^,^8^ However, these studies focus mainly on virus sampled from the nasopharynx, while emerging data suggest that virus sampled from different respiratory compartments (such as saliva or exhaled breath) has different viral load kinetics.^9^,^12^ Furthermore, many of these studies were cross-sectional, where testing was triggered by symptom onset, and comparisons of viral kinetics become difficult because of the impact of the epidemic growth rate.^13^ Thus, these studies will have missed the early stages of viral replication. Variation in viral load will also not be distinguishable from variation in viral load between individuals in these studies.^14^ Data syntheses conducted early during the COVID-19 pandemic attempting to summarise these data will have the same limitations.^15 16^

Since 2020, emerging studies have repeatedly tested individuals throughout the course of SARS-CoV-2 infection, making it possible to separate individual viral load kinetics from the between-individual variation in viral load. These studies re also present data by key covariables, such as host age, immune status, vaccination status and the infecting SARS-CoV-2 VOC.^17 18^ Additionally, human challenge models have allowed for estimation of viral load kinetics in the early exposure, pre-symptomatic period of infection.^5 19^ Finally, complex mechanistic modelling methods have been developed to allow for estimates of viral load trajectories for each individual, accounting for different covariates.^17 20^ We therefore aim to perform a systematic review and meta-analysis of studies that measure longitudinal SARS-CoV-2 viral load over the course of infection. We will present viral load kinetics across different respiratory compartments based on the available data; how this is associated with different measures of infectiousness; and how different covariates, such as age, exposure history and vaccination history, may affect viral load kinetics in these compartments. SARS-CoV-2 RNA viral loads isolated from the respiratory tract are much higher than from other anatomical sites, such as peripheral blood, stool, urine and ocular secretions. Samples from these other sites often have RNA viral loads that are usually incompatible with the presence of infectious virus. Such specimens are not considered relevant for airborne transmission, and therefore, we will concentrate on viral load from compartments within the respiratory tract. Finally, we will summarise the key mathematical models that have been used in the literature to estimate individual-level viral load trajectories, using data from their respective studies. We anticipate that our findings will demonstrate the importance of linking information on participant characteristics, symptom profile and infecting variants with prospective sampling from different respiratory compartments in relation to host infectiousness.

## Methods

### Protocol design and registration

This protocol is reported in accordance with the Preferred Reporting Items for Systematic Reviews and Meta-Analyses (PRISMA-P) reporting guidelines.^21^ The study protocol is registered with the International Registration of Systematic Reviews (PROSPERO ID: CRD42023385315). Data collection for this work is projected to be completed by August 2025, but the search may be updated to include important emerging work or to update the meta-analysis.

### Eligibility criteria

We aim to retrieve all English-language research articles reporting longitudinal RNA viral load from different respiratory compartments for SARS-CoV-2. We expect studies to typically include individual-level reports of RNA viral load over time in the upper respiratory tract and or other specimens, such as from exhaled breath. Studies with children will be included.

We will exclude review papers, animal studies, studies on environmental sampling, case reports and case series with less than five participants because of reporting bias and papers where there is no anchoring time that can be used to impute time of infection, whether that be date of PCR positive, symptom onset or date of hospital admission.

### Information sources

We will systematically search major databases, including MEDLINE and EMBASE using search strategies developed by an academic librarian (please see online supplemental appendices), from each database’s inception to date, which may be updated in line with ongoing work from the literature. We will also manually screen the references of included original studies to obtain additional studies.

### Search strategy

A Boolean search strategy will be used with a combination of subject headings specific to each database and relevant text words to search systematically for relevant studies. An asterisk will be used for abridged terminology. Search terms have been created in consultation with the lead author (DP) and an academic librarian (PD) to ensure that all relevant search terms are included and to develop a successful search strategy for each source of information and, therefore, to enhance the transparency and comprehensiveness of the review, as shown in online supplemental appendix 1. Search terms were developed from reviewing relevant research and have been piloted several times to refine their sensitivity and specificity before the searches are conducted.

### Selection process

All items identified using our search strategy will be assessed for eligibility through title and abstract screening, followed by a full-text screening, carried out by a minimum of two researchers independently.

### Data items

RNA viral load will be defined as either a value in genome copy number or a Ct value of an uncalibrated PCR assay. For human challenge studies, the time since the infection will be recorded; for real-world studies, we will report the time since symptom onset (or in asymptomatic subjects, time since a first positive test) from any sampling site. For each study, we will first record the following:

1. Name of first or corresponding author and date of publication.
2. Study duration and geographic location of study to infer what variant of SARS-CoV-2 may have been circulating if sequencing had not been performed.
3. Sample size.
4. Age.
5. Sex.
6. Ethnicity breakdown.
7. Whether the study was in adults or in children. Children and young people by and large did not develop severe COVID-19 but were considered a potential reservoir for infection, with educational consequences, such as school closures. RNA viral load kinetics will vary according to age.
8. Whether the study participants were mainly healthy individuals (such as infected healthcare workers) or immunosuppressed individuals, since immunosuppressed individuals are known to shed virus for longer periods.
9. Compartments that measured viral RNA (upper respiratory tract (subdivided into nose (and more subdivided compartments such as anterior nares, where possible), nasopharynx and oropharynx if data available), saliva, exhaled breath).
10. Modelling methods used to longitudinally map out RNA viral loads (see example in online supplemental appendix 2).
11. Whether the study was a real-world study or a human challenge.

Where presented, we will record RNA viral loads over time on an individual level if they are presented as either numerical values in tables, figures or in RNA viral load versus time plots. Diagnostic test results, as well as duration of positivity of virus culture, culture method, rapid antigen tests and immunology, will also be recorded if data are present. For example, if studies investigate quantitative virus infectivity as assessed by focus forming assay (FFA) or 50% tissue culture infectious dose (TCID 50) and fit our inclusion criteria, we will record this data. Where possible, numerical values will be copy-pasted directly into a comma-separated value format from the source, whereas tabulated numerical values contained in images will be extracted using https://extracttable.com/. RNA viral loads (and lateral flow assay (LFA) results/virus culture results/immunology) reported in plots will be extracted using WebPlotDigitizer (Pacifica, CA: Ankit Rohatgi).^22^ In addition, we will extract the following patient-level covariables per individual where presented within the manuscript.

1. Whether the study was a human challenge study, performed in a superficial setting or an observational cohort of participants that were routinely infected in their daily lives.
2. Clinical symptoms, timing of symptoms and their severity throughout infection.
3. Age.
4. Sex.
5. Ethnicity.
6. Vaccination status (including vaccination regimen and time since last vaccine if available) and any previous infections.
7. Whether any antiviral therapy/other treatment was initiated throughout the course of infection.
8. Need for and days of hospitalisation/oxygenation if relevant.
9. Need for and days of mechanical ventilation if relevant.
10. Whether the patient died and the time to death.
11. Whether there was a recording of transmission from an index participant to a contact within a household or other settings.

A quality control check will be performed for each paper by an independent reviewer who was not responsible for extracting the raw data.

### Quality assessment

We will conduct two quality assessments; first, on the quality of the longitudinal studies themselves and, second, on the quality of RNA viral load data collected. To report the former, we will use a tool developed and validated by Tooth and colleagues developed specifically for longitudinal studies, which consists of a checklist of 33 criteria.^23^ Quality of RNA viral load data reporting is required, because many studies only report Ct values, which indirectly provide a quantitative PCR value not interchangeable between assays and can be affected by the gene target or targets being assayed, the nucleic acid extraction system and PCR amplification biochemistry.^24^ Consequently, we will record the PCR platform used, gene target and non-SC2 control targets as appropriate.

Quality of RNA viral load reporting will be graded as follows:

1: RNA viral load reported in PCR Ct but primers not reported so an assumed calibration curve from another source will be used to convert RNA viral load in copies/mL.

2: RNA viral load reported in PCR Ct but primers reported and a calibration curve from another source can be used to convert viral load in copies/mL.

3: RNA viral load reported in copies/mL or a calibration curve reported in the manuscript, allowing conversion of PCR Ct to copies/mL.

4: RNA viral load reported in copies/mL or a calibration curve reported in the manuscript, and LFA (rapid antigen test) or virus culture is also reported.

5: RNA viral load reported in copies/mL or a calibration curve reported in the manuscript, and LFA and virus culture are also reported.

### Data synthesis

#### Summary statistics

For eligible studies, we will calculate the mean duration of viral emission and 95% CI if the data are presented. To estimate a pooled effect size, we will apply a random-effects model. Then, we will generate forest plots to show the detailed representation of all studies, based on the effect size and 95% CI. If not reported, we will derive means and standard errors from sample size, median, IQR and minimum and maximum values.^25^ The time from anchoring to peak viral load, as well as the longest duration of viral load in each compartment, will also be tabulated. Heterogeneity between studies will be quantified by the I^2^ index and Cochran’s Q test. We will use meta-regression to assess the effect of potential moderators on the pooled effect size; p<0.05 will be considered to be significant. In this analysis, we will also present periods during which LFA, rapid antigen tests and virus culture results are most likely to be positive. Human challenge studies will be analysed separately to real-world studies.

### Statistical analysis

We will use a multilevel survival analysis (clustered by study), with the dependent variable being time to viral clearance, defined as the time of the first sample falling below the limit of quantification following the last above limit of quantification sample (note if samples become positive after being negative, the earlier negative sample will be discounted). The final sample is above the limit of quantification, and the case will be treated as censored. A multivariable analysis will then be undertaken in *R*, accounting for patient covariables (age, sex, ethnicity, vaccination status, previous infections), viral covariates (variants) sampling compartment and antiviral therapies. Missing covariate data may be handled using multiple imputation, depending on the degree of missingness (10% or more or depending on the pattern of missingness in the covariate data). Sensitivity analysis may also be performed, based on viral loads generated from the different assays and PCR targets used in each study.

### Mechanistic data synthesis

Studies included in our systematic review are expected to construct mathematical models that estimate differences in viral load kinetics, accounting for both individual- and group-level variation in viral load. They are also expected to adjust for a changing background of host characteristics, possibly over time, depending on the cohort study, to find how different SARS-CoV-2 VOCs may peak at different timepoints, as well as other factors that may have an influence on viral load, such as age and vaccination status. We aim to summarise the key mechanistic models adopted during different phases of the COVID-19 pandemic (early in 2020, before reinfection or vaccination, to 2023, where different exposures to previous infection, different VOCs and vaccination regimens are present).^17 26^ An example of a classic within-host viral load kinetic model is given in Appendix 2. Other examples may include models that integrate host/pathogen interaction to reconstruct RNA viral load kinetic profiles and metrics of interest.

Details of any sensitivity analysis with alternative model fits (including alternative model structures and observation distributions), adjustment for different characteristics and simulations run will be recorded. The risk of meta-bias will be assessed using funnel plots and Egger’s test. Strength of the body of evidence will be assessed using the Grading of Recommendations Assessment, Development and Evaluation (GRADE) criteria.

## Ethics and dissemination

No ethical approval will be needed since the data will be obtained from published articles and reports. Given the quantity of methods reviewed and multiple analyses outlined, more than one output may be generated from this piece of work. Should the data allow, we may also seek to perform individual patient-level meta-analyses of the data for some specific outcomes. We will prepare one/multiple manuscripts for publication in peer-reviewed journals and disseminate the findings and results of our work at relevant conferences.

## supplementary material

10.1136/bmjopen-2024-085127 online supplemental file 1
