# Supplementary material for: Defining within-host SARS-CoV-2 RNA viral load kinetics during acute COVID-19 infection within different respiratory compartments and their respective associations with host infectiousness: a protocol for a systematic review and meta-analysis
Source: BMJ Open. 2024 Dec 2;14(12):e085127. doi: 10.1136/bmjopen-2024-085127 (PMC11624818; doi:10.1136/bmjopen-2024-085127)
Supplement: online supplemental file 1 [file bmjopen-14-12-s001.docx]

**Supplementary materials for: Defining within-host SARS-CoV-2 RNA viral load kinetics during acute illness in different respiratory compartments and its association with infectiousness: a protocol for a systematic review and meta-analysis**

Daniel Pan^1,2,3,4^^,5,6^, Christopher A Martin^1,2,3,4^, Joshua Nazareth^1,2,3,4^, Shirley Sze^7^, Amani Al-Oraibi^1,3,8^, Mayuri Gogoi^1,3^, Natalia Grolmusova^2,3,4^, Pip Divall^9^, Jonathan Decker^3^, Eve Fletcher^3,4^, Caroline Williams^3,4,10^, James Hay^5^, Rebecca F Baggaley^1,4,19^, Anne L. Wyllie^11^, Iain Stephenson^2^, Erol A Gaillard^3,4,13^, Laura B Nellums^14^, Tristan W Clark^15,16,17^, Jonathan S Nguyen-Van-Tam^8^, Benjamin Cowling^6,18^, T. Déirdre Hollingsworth

^5,19*^, Laura J Gray^4,20, 21*^, Michael R Barer^3,10*^, Manish Pareek^1,2,3,4,21*^

^1^ Development Centre for Population Health, University of Leicester, Leicester, UK

^2^Department of Infectious Diseases and HIV Medicine, Leicester Royal Infirmary, University Hospitals of Leicester NHS Trust, Leicester, UK

^3^Department of Respiratory Sciences, University of Leicester, Leicester, UK

^4^NIHR Leicester Biomedical Research Centre, Leicester, UK

^5^Oxford Big Data Institute, Li Ka Shing Institute of Health Information and Discovery, University of Oxford, Oxford, UK

^6^WHO Collaborating Centre for Infectious Disease Epidemiology and Control, School of Public Health, Li Ka Shing Faculty of Medicine, The University of Hong Kong, Hong Kong, China

^7^Department of Cardiovascular Sciences, University of Leicester, Leicester, UK

^8^Lifespan and Population Health, University of Nottingham School of Medicine, Nottingham, UK

^9^Education Centre Library, Glenfield and Leicester Royal Infirmary, University Hospitals of Leicester NHS Trust, Leicester, UK

^10^Department of Clinical Microbiology, University Hospitals of Leicester NHS Trust, Leicester, UK

^11^Department of Epidemiology of Microbial Diseases, Yale School of Public Health, New Haven, CT, USA

^13^Department of Paediatric Respiratory Medicine, University Hospitals of Leicester NHS Trust, Leicester, UK

^14^College of Population Health, University of New Mexico, Albuquerque, New Mexico, United States

^15^Clinical and Experimental Sciences, Faculty of Medicine, University of Southampton, UK

^16^Department of Infection, University Hospital Southampton NHS Foundation Trust, UK

^17^NIHR Southampton Biomedical Research Centre, UK

^18^Laboratory of Data Discovery for Health Limited, Hong Kong Science and Technology Park, New Territories, Hong Kong, China

^19^NDM Centre for Global Health Research, Nuffield Department of Medicine, University of Oxford, UK

^20^Department of Population Health Sciences, University of Leicester, UK

^21^NIHR Applied Research Collaborative East Midlands

* Joint senior authors

Corresponding author: Professor Manish Pareek

Email: [manish.pareek@leicester.ac.uk](mailto:manish.pareek@leicester.ac.uk)

**Appendix 1: Search strategy for identifying publications reporting SARS-CoV-2 RNA viral load**

MEDLINE(R) ALL <1946 to December 05, 2022>

1 SARS-CoV-2/ or COVID-19/ 309455

2 (corona* adj1 (virus* or viral*)).ti,ab,kw,kf. 11771

3 (CoV not (Coefficien* or "co-efficien*" or covalent* or Covington* or covariant* or covarianc* or "cut-off value*" or "cutoff value*" or "cut-off volume*" or "cutoff volume*" or "combined optimi?ation value*" or "central vessel trunk*" or CoVR or CoVS)).ti,ab,kw,kf. 224990

𝑔(𝑡) =

⎩ ⎪ ⎪ ⎨

4 (coronavirus* or 2019nCoV* or 19nCoV* or "2019 novel*" or Ncov* or "n-cov" or "SARS-CoV-2*" or "SARSCoV-2*" or SARSCoV2* or "SARS-CoV2*" or "severe acute respiratory syndrome*" or COVID*2).ti,ab,kw,kf. 682012

5 or/1-4 696844

6 limit 5 to yr="2020-Current" 668312

7 6 not (letter or historical article or comment or editorial or news).pt. not (Animals/ not humans/) 574110

8 Virus Shedding/ 13757

9 ((viral or virus) adj3 shed*).mp. [mp=ti, ab, hw, tn, ot, dm, mf, dv, kf, fx, dq, bt, nm, ox, px, rx, ui, sy] 21089

10 RNA, Viral/ 162972

11 (viral adj3 clearance).mp. [mp=ti, ab, hw, tn, ot, dm, mf, dv, kf, fx, dq, bt, nm, ox, px, rx, ui, sy] 19510

12 viral load/ 128855

13 ((viral or virus) adj3 kinetic*).mp. [mp=ti, ab, hw, tn, ot, dm, mf, dv, kf, fx, dq, bt, nm, ox, px, rx, ui, sy] 6024

14 human challenge.mp. 960

15 or/8-14 306838

16 infectiv*.mp. [mp=ti, ab, hw, tn, ot, dm, mf, dv, kf, fx, dq, bt, nm, ox, px, rx, ui, sy] 250804

17 infectious*.mp. 758291

18 contagious*.mp. 27246

19 transmiss*.mp. 1380166

20 live virus.mp. 5278

21 viral culture*.mp. 4446

22 or/16-21 2256443

23 7 and 15 and 22 7645

24 exp severe acute respiratory syndrome coronavirus 2/ or coronavirus disease 2019/ or experimental coronavirus disease 2019/ 499000

25 (corona* adj1 (virus* or viral*)).ti,ab,kw. 10607

26 (CoV not (Coefficien* or co-efficien* or covalent* or covington or covariant* or covarianc* or "cut-off value*" or "cutoff value*" or "cut-off volume*" or "cutoff volume*" or "combined optimi?ation value*" or "central vessel trunk" or CoVR or CoVS)).ti,ab,kw. 198477

27 (coronavirus* or 2019nCoV* or 19nCoV* or "2019 novel*" or Ncov* or "n-cov" or "SARS-CoV-2*" or "SARSCoV-2*" or SARSCoV2* or "SARS-CoV2*" or "severe acute respiratory syndrome*" or COVID*2).ti,ab,kw. 676264

28 or/24-27 709741

29 limit 28 to yr="2020-Current" 681320

30 29 not (letter or editorial).pt. not (nonhuman/ not human/) not (conference abstract or conference paper or conference proceeding or "conference review").pt. 524355

31 ((viral or virus) adj3 shed*).mp. 21089

32 virus shedding/ 13757

33 virus RNA/ 91532

34 (viral adj3 clearance).mp. [mp=ti, ab, hw, tn, ot, dm, mf, dv, kf, fx, dq, bt, nm, ox, px, rx, ui, sy] 19510

35 viral clearance/ 11714

36 virus load/ 101725

37 ((viral or virus) adj3 kinetic*).mp. 6024

38 human challenge.mp. 960

39 or/31-38 217066

40 infectiv*.mp. 250804

41 infectious*.mp. 758291

42 contagious*.mp. 27246

43 transmiss*.mp. 1380166

44 live virus.mp. 5278

45 virus culture/ or viral culture*.mp. 15874

46 or/40-45 2264650

47 30 and 39 and 46 5269

48 23 use medall 2266

49 47 use oemezd 4357

50 48 or 49 6623

Search Name: EMBASE

Date Run: 06/12/2022 17:34:42

ID Search Hits

#1 MeSH descriptor: [SARS-CoV-2] explode all trees 1187

#2 MeSH descriptor: [COVID-19] explode all trees 2553

#3 (corona* near/1 (virus* or viral*)) 418

#4 (CoV not (Coefficien* or "co-efficien*" or covalent* or Covington* or covariant* or covarianc* or "cut-off value*" or "cutoff value*" or "cut-off volume*" or "cutoff volume*" or "combined optimi?ation value*" or "central vessel trunk*" or CoVR or CoVS)) 909

#5 (coronavirus* or 2019nCoV* or 19nCoV* or "2019 novel*" or Ncov* or "n-cov" or "SARS-CoV-2*" or "SARSCoV-2*" or SARSCoV2* or "SARS-CoV2*" or "severe acute respiratory syndrome*" or COVID*2) 10496

#6 {or #1-#5} with Publication Year from 2020 to 2022, in Trials 10955

#7 MeSH descriptor: [Virus Shedding] explode all trees 223

#8 ((viral or virus) near/3 shed*) 921

#9 MeSH descriptor: [RNA, Viral] explode all trees 2040

#10 viral near/3 clearance 611

#11 MeSH descriptor: [Viral Load] explode all trees 2572

#12 ((viral or virus) near/3 kinetic*) 359

#13 "human challenge" 122

#14 {or #7-#13} 5492

#15 infectiv* 7610

#16 infectious* 26729

#17 contagious* 286

#18 transmiss* 12675

#19 "live virus" 112

#20 "viral culture*" 117

#21 {or #15-#20} 43681

#22 #6 and #14 and #21 119

**Appendix 2**

A typical within-host viral load kinetics model assumes a linear increase, a changepoint representing the peak of viral load and a linear decrease on the cycle threshold scale (log viral load scale). Given the relationship between Ct values and viral load, this represents an exponential increase and decrease (with two separate rates) on the viral load scale. There are multiple ways to capture this process mathematically, but the most common is to describe a piecewise linear function with parameters representing the timing of and rate of virus growth and decline.

The Ct kinetics model is given by:


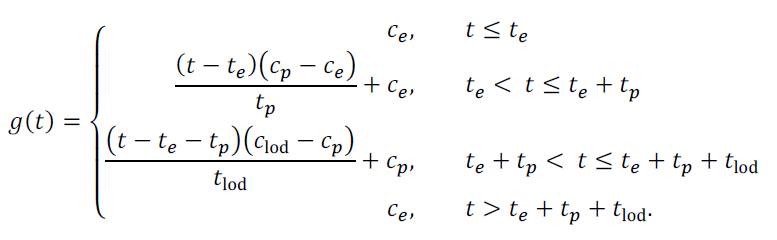


where 𝑡! is time the individual was exposed, 𝑡" is the time at which the individual’s Ct

trajectory peaks, 𝑡&'( is the time at which the trajectory hits the limit of detection, 𝑐! is the

theoretical Ct value at exposure, 𝑐" is the Ct value at the peak of the trajectory, and 𝑐lod is

the theoretical value at which a near-perfect PCR machine would no longer detect any virus.

The parameters *ce, cp*, *tp*, *te* and *tlod* are typically estimated using maximum likelihood or Bayesian methods linking the model trajectory to observed data through a likelihood function. A typical approach is to estimate separate parameter values for each individual in the dataset, using a hierarchical model to estimate both individual-level variation and population-average trajectories, as well as the contribution of key covariates such as vaccination status or variant to these parameters. *C_lod* is usually a fixed parameter based on the assay used. The parameter *t_e*, the time of exposure, is always unobserved but can also be jointly estimated when fitting the model relative to the timing of first positive test, timing of symptom onset or time of lowest observed Ct value.
