# Supplementary material for: Defining within-host SARS-CoV-2 RNA viral load kinetics during acute COVID-19 infection within different respiratory compartments and their respective associations with host infectiousness: a protocol for a systematic review and meta-analysis
Source: BMJ Open. 2024 Dec 2;14(12):e085127. doi: 10.1136/bmjopen-2024-085127 (PMC11624818; doi:10.1136/bmjopen-2024-085127)
Supplement: Supplementary file 2 [file bmjopen-2024-085127.reviewer_comments.pdf]

PEER REVIEW HISTORY

BMJ Open publishes all reviews undertaken for accepted manuscripts. Reviewers are asked to complete a checklist review form and are provided with free text boxes to elaborate on their assessment. These free text comments are reproduced below.

ARTICLE DETAILS

Title (Provisional)

Defining within-host SARS-CoV-2 RNA viral load kinetics during acute illness in different respiratory compartments and its association with infectiousness: a protocol for a systematic review and meta-analysis

Authors

Pan, Daniel; Martin, Christopher A; Nazareth, Joshua; Sze, Shirley; Al-Oraibi, Amani; Gogoi, Mayuri; Grolmusova, Natalia; Divall, Pip; Decker, Jonathan; Fletcher, Eve; Williams, Caroline; Hay, James; Baggaley , Rebecca F.; Wyllie, Anne L.; Stephenson, Iain; Gaillard, Erol; Nellums, Laura B; clark, tristan William; Van-Tam, Jonathan Nguyen; Cowling, Benjamin J; Hollingsworth, Deirdre T; Gray, Laura; Barer, Michael; Pareek, Manish

---

VERSION 1 - REVIEW

**Reviewer**	**1**
**Name**	**Guedj, Jeremie**
**Affiliation**	**Universite de Paris, IAME, INSERM**
**Date**	**20-Feb-2024**
**COI**	**none**

The protocol mentions "mechanistic models" but the models proposed are not really mechanistic and are rather descriptive. The use of genuine mechanistic models integrating host/pathogen interaction could be helpful (at least as a sensitivity analysis) to reconstruct more precisely viral kinetic profiles and estimate the metrics of interest.

Also the authors focus on RNA viral load, which is important, but they should consider applying the same strategy to quantitative virus infectivity (as assessed by FFA or TCID50), which is another important marker that has been much less studies and could be relevant to quantify infectiousness.

**Reviewer**	**2**
**Name**	**Ghoddoosi-Nejad, Djavad**
**Affiliation**	**Birjand University of Medical Sciences**

Date **02-Aug-2024**

COI **none.**

Der authors

I read and enjoyed your prestigious work on this MS. Obviously there have been a lot of efforts and precision effort on this MS. Wish you the best.

VERSION 1 - AUTHOR RESPONSE

Reviewer: 1

Prof. Jeremie Guedj, Universite de Paris

Comments to the Author:

The protocol mentions "mechanistic models" but the models proposed are not really mechanistic and are rather descriptive. The use of genuine mechanistic models integrating host/pathogen interaction could be helpful (at least as a sensitivity analysis) to reconstruct more precisely viral kinetic profiles and estimate the metrics of interest.

Thank you. We agree and have added an extra statement describing collection of genuine mechanistic models:

'Other examples may include models that integrate host/pathogen interaction to reconstruct RNA viral load kinetic profiles and metrics of interest.'

Also the authors focus on RNA viral load, which is important, but they should consider applying the same strategy to quantitative virus infectivity (as assessed by FFA or TCID50), which is another important marker that has been much less studies and could be relevant to quantify infectiousness.

Thank you. We agree and have expanded this statement within our methods:

'Where presented, we will record RNA viral loads over time on an individual level if they are presented as either numerical values in tables, figures, or in RNA viral load vs time plots. Diagnostic test results, as well as duration of positivity of virus culture, culture method, rapid antigen tests and immunology will also be recorded if data are present. For example, if studies look at quantitative virus infectivity as assessed by FFA or TCID 50, and fit our inclusion criteria, we will record this data.'

Reviewer: 1
Competing interests of Reviewer: none

****

Reviewer: 2

Dr. Djavad Ghoddoosi-Nejad, Birjand University of Medical Sciences

Comments to the Author:

Der authors
I read and enjoyed your prestigious work on this MS. Obviously there have been a lot of efforts and precision effort on this MS. Wish you the best.

Thank you very much.

Reviewer: 2
Competing interests of Reviewer: none.

****
